# New atrial arrhythmia occurrence in single chamber implantable cardioverter defibrillator patients: A real‐world investigation

**DOI:** 10.1111/jce.15790

**Published:** 2022-12-30

**Authors:** Divyang Patel, Archana Rao, Paul A. Friedman, Abhishek J. Deshmukh, Jeff Lande, Jeffrey A. Murphy, Mark L. Brown, Daniel R. Lexcen, Bruce L. Wilkoff

**Affiliations:** ^1^ Division of Cardiology Sentara Heart Hospital Norfolk Virginia USA; ^2^ Institute of Cardiovascular Medicine and Science at Liverpool Heart and Chest Hospital Liverpool Liverpool UK; ^3^ Department of Cardiovascular Medicine Mayo Clinic Rochester Minnesota USA; ^4^ Medtronic Inc Mounds View Minnesota USA; ^5^ Department of Cardiovascular Medicine Heart Vascular and Thoracic Institute Cleveland Clinic Cleveland Ohio USA

**Keywords:** anticoagulation, atrial fibrillation, cardiac implantable electronic device, device algorithm, implantable cardioverter defibrillator, single chamber

## Abstract

**Introduction:**

A current limitation of single chamber implantable cardioverter defibrillators (ICDs) is the lack of an atrial lead to reliably detect atrial fibrillation (AF) episodes. A novel ventricular based atrial fibrillation (VBAF) detection algorithm was created for single chamber ICDs to assess R‐R variability for detection of AF.

**Methods:**

Patients implanted with Visia AF™ ICDs were prospectively enrolled in the Medtronic Product Surveillance Registry from December 15, 2015 to January 23, 2019 and followed with at least 30 days of monitoring with the algorithm. Time to device‐detected daily burden of AF ≥ 6 min, ≥6 h, and ≥23 h were reported. Clinical actions after device‐detected AF were recorded.

**Results:**

A total of 291 patients were enrolled with a mean follow‐up of 22.5 ± 7.9 months. Of these, 212 (73%) had no prior history of AF at device implant. However, 38% of these individuals had AF detected with the VBAF algorithm with daily burden of ≥6 min within two years of implant. In these 80 patients with newly detected AF by their ICD, 23 (29%) had a confirmed clinical diagnosis of AF by their provider. Of patients with a clinical diagnosis of AF, nine (39%) were newly placed on anticoagulation, including five of five (100%) patients having a burden >23 h.

**Conclusions:**

Continuous AF monitoring with the new VBAF algorithm permits early identification and actionable treatment for patients with undiagnosed AF that may improve patient outcomes.

## 1 INTRODUCTION

Atrial fibrillation (AF), the most common arrhythmia in practice, affects 30–40 million people worldwide and is a major cause of morbidity and mortality.^1^ The management of AF includes an anticoagulation strategy to prevent cerebrovascular accidents (CVAs), a rate control approach in patients who are relatively asymptomatic, and/or a rhythm control strategy in selected patients with the combination of direct current cardioversion, anti‐arrhythmics, and/or catheter‐based ablation.^2^, ^3^ However, each of these treatment approaches relies on adequately detecting AF, which is difficult given that a significant proportion of patients may be asymptomatic.^4^, ^5^, ^6^

Patients with cardiac implantable electronic devices (CIEDs) are at high risk for complications from AF, such as CVAs or heart failure exacerbations, as these patients are usually older and have a greater number of comorbidities including heart failure, diabetes, and ischemic disease.^7^ The ASSERT trial first enrolled 2580 patients who were age 65 and older with recently implanted defibrillators and pacemakers with no previous history of atrial tachy‐arrhythmias (AT) and found evidence of subclinical AT lasting longer than 6 min in 34.7% of patients at mean follow‐up of 2.5 years.^8^ Those with subclinical AT had a higher rate of stroke (4.2% vs. 1.7%) during the follow‐up period than those patients who did not experience any tachyarrhythmias.^8^ This finding was later confirmed by the SOS AF project which included a pooled analysis of AF in greater than 10 000 patients implanted with CIEDs.^9^

Because single chamber implantable cardioverter defibrillators (ICDs) do not have an atrial lead, these systems lack the ability to detect AF. Therefore, some providers have implanted dual chamber ICDs in patients with a previous history of AF to better discriminate episodes of AT from ventricular arrhythmias. In fact, data from the National Cardiovascular Data Registry (NCDR) ICD registry by Matlock et al., found that electrophysiologists were significantly more likely to implant a dual chamber ICD compared to single chamber ICD in patients with a history of AF likely given the need to avoid inappropriate shocks.^10^ However, Friedman et al. showed that with newer and better algorithms the rate of inappropriate shocks are no different in patients with single chamber ICDs compared to dual chamber ICDs, although the latter adds additional cost.^11^ Additional risks of an atrial lead include, but are certainly not limited to, increasing rates of mechanical and infectious complications.^12^

A novel ventricular‐based atrial fibrillation algorithm (VBAF) has been developed for single chamber ICDs that assesses consecutive R‐wave (R‐R) variability as a surrogate for episodes of AF. The algorithm has been previously described and studied using expert Holter recordings from multiple different sources and found to have high sensitivity and specificity for AF detection (95 percent sensitive; 99.5 percent specificity for gross duration of AF of at least 6 min in length).^13^ The objective of the current study is to report our findings of VBAF‐reported AF in a real world multi‐center cohort and the clinical actions taken with the information provided by the VBAF algorithm in patients with single chamber ICDs.

## 2 METHODS

### 2.1 Study design and patients

This prospective, multicenter, observational study was conducted within the Medtronic Product Surveillance Registry (PSR), an active global post‐market surveillance platform designed to further evaluate safety and effectiveness of products in “real‐world” clinical practice following commercial release (Clinicaltrials.gov number: NCT01524276). The protocol was approved by an institutional review board or an ethics committee at each participating center as applicable per local regulations. All patients provided informed consent. Patients were eligible for enrollment if they were implanted with a VBAF detection algorithm enabled device (Visia AF ICD with TruAF™ algorithm, Medtronic Inc). Prospective enrollment was defined as before the implantation procedure or up to 30 days postimplantation. There were no protocol exclusions regarding patient age, sex, or gender. Enrollment occurred at 60 clinical centers in the United States and Europe from December 2015 to January 2019. Detailed baseline demographic and clinical characteristics were obtained.

After successful implantation, patients were followed according to routine clinical practices until patient exit, death or the end of device performance, for example, explant. Patient and device status were reported at follow‐up visits which were expected to occur approximately every 6 months per standard of care procedures. Device data through remote transmission to the Medtronic CareLink™ network was recorded including daily AF burden. Based on the purpose of this study, patients were included in the endpoint analysis if they were (1) implanted with a VBAF‐capable single chamber ICD with the algorithm activated, (2) with no AF history, and 3) with at least 30 days of CareLink remote monitoring.

### 2.2 Endpoints

The primary endpoint was the incidence of device‐detected AF 6 or more minutes in duration at 24 months. The VBAF detection algorithm also detects atrial flutter and AT that conducts to the ventricle in an irregular manner. Thus, AF within this study refers to atrial fibrillation, atrial tachycardia or atrial flutter as detected by the device. Per the algorithm, a minimum AF episode duration of 6 min was required for detection given existing evidence which suggested that AF lasting 6 min or longer was associated with a higher risk of stroke or embolism in CIED patients.^8^, ^9^

The time to first device‐detected daily burden of AF ≥ 6 min, ≥6 h, and ≥23 h was summarized. In patients whom no clinical action was taken, AF episodes in patients without a history of AF were adjudicated by an independent panel of electrophysiologists, who were blinded to clinical actions, to determine which patients had true AF episodes recorded by the ICD. AF adjudication was at the patient level and not episode level. This means that if a consensus of AF was made for at least one episode, then the patient was considered to have device detected AF. Otherwise, the patient was deemed not to have it. Episodes were adjudicated by two reviewers. Episodes not achieving consensus were sent to a third reviewer for final classification. Each reviewer was blinded to what the other reviewers classified for each patient/episode.

### 2.3 Statistical analysis

Descriptive statistics are expressed as frequencies and percentages, or as means ± standard deviations, as appropriate. *p* values are two‐sided and <0.05 is considered statistically significant. The cumulative incidence of AT/AF is estimated using Kaplan–Meier method. Time 0 is defined as the ICD implant date for the survival analysis. Patients without an endpoint during follow‐up were censored at the date of last patient contact or the deactivation of the device. All analyses used the SAS Studio (v. 3.8; SAS Institute).

## 3 RESULTS

### 3.1 Enrollment and baseline characteristics

**Figure 1:**
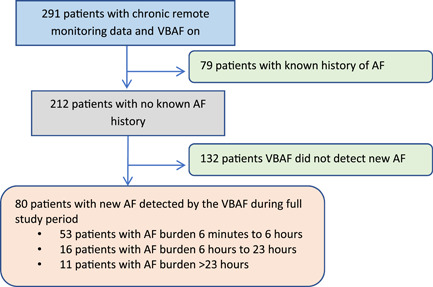
Patient accountability flow diagram

Between December 2015 and January 2019, 291 patients underwent successful implantation of a VBAF‐capable single chamber ICD and were prospectively enrolled in the registry. A total of 212 patients met the inclusion criteria for the analysis after excluding 79 patients with a history of AF. A summary of study patient status is presented in Figure 1.

Baseline patient characteristics of the 212 patients included in the analysis are displayed in Table 1. The mean age was 60 ± 13 years, majority (66%) were males with New York Heart Association (NYHA) class II symptoms (54%). The mean CHADS_2_‐VAS_C_ score was 3.3 ± 1.6 and the mean left ventricular ejection fraction (LVEF) was 33 ± 13%. The mean follow‐up time was 22.8 ± 7.6 months.

**Table 1:** Baseline demographics

Subject characteristic	Patients with no AF history, chronic remote monitoring data, and VBAF algorithm activated (*N* = 212)
Male	139 (66%)
Age (years)	60 ± 13
LVEF (%)	33 ± 13
CHA_2_DS_2_‐VASc score	3.3 ± 1.6
Secondary prevention	29 (16%)
NYHA class	
None	6 (6%)
I	13 (14%)
II	51 (54%)
III	23 (24%)
IV	1 (1%)
Ischemic cardiomyopathy	88 (46%)
Congestive heart failure	96 (50%)
Coronary artery disease	87 (46%)
Hypertension	116 (61%)
Myocardial infarction	68 (35%)
Any valve surgery	7 (4%)
Coronary artery intervention	59 (31%)
Previous implantable CIED	50 (24%)
AV Block	12 (7%)
Left bundle branch block	8 (5%)

*Note*: Values are means ± standard deviation or *n* (%). Percentages are adjusted for missing values. CHA2DS2‐VASc score calculates stroke risk for patients with atrial fibrillation using the following demographics and medical history: Congestive heart failure, Hypertension, age ≥ 75 (2 points), diabetes, stroke (2 points), vascular disease, age 65 to 74, and sex category (female).

Abbreviations: AV, atrioventricular; CIED, cardiovascular implantable electronic device; LVEF, left ventricular ejection fraction; NYHA, New York Heart Association.

### 3.2 Incidence of device‐detected atrial fibrillation at follow‐up

**Figure 2:**
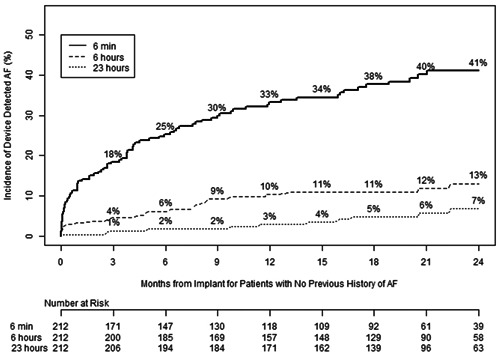
Incidence of device detected atrial fibrillation. The Kaplan–Meier estimate for cumulative incidence of device detected atrial fibrillation (AF) of ≥6 min (solid line), >6 h (larger dotted line), and >23 h (smaller dotted line) in patients without history of AF is shown.

The total incidence of AF in patients with no history of AF was 38% (80/212 patients). In the 80 patients with newly detected AF during the study, the median (interquartile range) of days from device implantation to first device detected AF was 104.0 (13.0–254.5) days. At 1‐year follow‐up after device implantation, the Kaplan–Meier rates of device‐detected AF in patients without a previous known history of AF with daily burden of ≥6 min, >6 h and >23 h were 33%, 10%, and 3%, respectively (Figure 2). Furthermore, at 2‐year follow‐up, the rates of device‐detected AF in the same cohort with a daily burden of ≥6 min, >6 h and >23 h were 41%, 13%, and 7%, respectively (Figure 2). All 80 patients' device detected episodes were reviewed by an independent episode review committee, until a true AF episode was discovered, and it was determined that true AF was documented in 100% of the >23 h, 75% of the >6 h and 40% in the >6 min AF burden cohorts.

### 3.3 AF confirmation, clinical symptoms, and actions taken

Of the 80 patients that were determined to have device‐detected AF, without a known previous history, with a burden >6 min, 23 patients (29%) were further evaluated for a clinical diagnosis, assessment of clinical symptoms, and to record actions taken in response to device detected AF. The average CHADS_2_‐VAS_C_ scores of these 80 patients was 3.57 ± 1.76. Overall, it was reported that clinicians used CareLink or CareAlert™ systems to confirm the device detected AF as a true clinical diagnosis of AF for the majority of patients evaluated (87%; 20/23). A total of 17 out of the 23 patients (74%) were asymptomatic, but for those that did report symptoms, the most common were palpitations (three patients) and dyspnea (three patients). Furthermore, of the 23 patients with a clinically evaluated diagnosis of AF, nine patients (39%) were newly initiated on oral anti‐coagulation, while two patients (8.7%) were newly started on antiplatelet therapy. Additionally, during the follow‐up period, 2 of the 23 patients (8.7%) were initiated on antiarrhythmic therapy while no patients underwent ablation for their AF.

A combined analysis of AF burden, CHA_2_DS_2_‐VAS_C_ scores, and actions taken are provided in Table 2. Clinical action was taken across the whole spectrum of CHA_2_DS_2_‐VAS_C_ scores while more clinical actions were taken for patients with higher AF burden: 45% for patients with >23 h burden, 43% for patients with burden between 6 and 23 h, and only 21% for patients with burden between 6 min and 6 h. Further, 100% (5/5) of the patients in the >23 h burden category were initiated on oral anti‐coagulants, compared to 17% (1/6) and 20% (2/10) of the 6 h to 23 h and 6 min to 6 h AF burden patients, respectively. Eighty percent (4/5) of the patients from the >23 h burden group had cardiovascular‐related healthcare utilization since their prior assessment, compared to 33% (2/6) for the 6 h to 23 h patients and 20% (2/10) for the 6 min to 6 h burden category. Only two patients had initiation or adjustment of their atrioventricular nodal blocking agent for rate control; both of these patients were in the >23 h burden category.

**Table 2:** Clinical actions taken across AF burden and CHA_2_DS_2_‐VASc

	Burden by CHA_2_DS_2_‐VASc score with clinical diagnosis					
Burden	CHA_2_DS_2_‐VASc score					Total
	≤2	3	4	5	≥6	
6 m to 6 h	11 (2)	15 (1)	9 (3)	8 (3)	4 (1)	47 (10) 21%
6 h to 23 h	1 (1)	4 (2)	4 (1)	3 (1)	2 (1)	14 (6) 43%
23 h or more	5 (3)	0	2 (1)	2	2 (1)	11 (5) 45%
Total	17 (6) 35%	19 (3) 16%	15 (5) 33%	13 (4) 31%	8 (3) 38%	72^a^ (21^b^) 29%

*Note*: (Clinical Diagnosis) %.

^a^ Eight of the 80 patients with no known history of AF but VBAF detected AF ≥ 6 min did not have all criteria to determine a CHADS‐VASc score.

^b^ Two of the 23 patients with a clinical diagnosis of AF did not have all criteria to determine a CHADS‐VASc score.

## 4 DISCUSSION

In this real‐world prospective registry, we report that a large proportion of patients with single chamber ICDs without a history of AF have newly diagnosed AF as detected by their device. This was confirmed clinically in 29% (23/80) of patients with device detected AF. While the VBAF specificity was not prospectively collected, it was observed that in patients with episodes ≥23 h the adjudication of saved device data could be used to confirm 100% of those episodes as true AF. Furthermore, 100% of patients with no known history of AF, but device detected AF with a burden of at least 23 h and a clinical diagnosis of AF, were initiated on oral anti‐coagulants. Our findings are important because we show that in the real world the VBAF algorithm detected AF and guided management, especially in those whose burden was ≥23 h.

Device‐detected AF has long perplexed electrophysiologists as there has been no consensus on the threshold duration of AF detected by the device that increases the risk of thromboembolism, especially in asymptomatic patients. Previous large studies including the ASSERT trial did show that patients with ≥6 min of CIED‐detected subclinical AT have higher risk of stroke compared to patients without device‐detected AT.^8^ However, there have been no prospective studies that have randomized patients with device‐detected AF to anticoagulation versus no anticoagulation. Therefore, the development of improved algorithms and devices that can better detect AF in CIED patients, as well as corresponding research on patient management, is essential.

Previous large studies that have looked at the importance of ICD or permanent pacemaker‐detected AF have found an incidence of 30%–45% over a mean follow‐up time of 1–3 years^8^, ^14^, ^15^ which is similar to our study where we found an AF rate of 38% (80/212) at 2 years, after single chamber ICD implantation. The main distinctions between our study and the aforementioned studies are that previous studies only examined short durations of AF and only included patients with dual chamber ICDs given the low sensitivity and specificity to detect AF in previous generation single chamber ICD devices.^8^, ^14^, ^15^ However, given the novel VBAF algorithm in these ICDs, the device was able to detect AF in CIED patients at similar rates compared to previous studies with dual chamber ICD patients. A study in patients with subcutaneous ICDs found a corrected incidence of 17.6%, which is lower than the rate in this study, likely driven by less comorbid and younger population who receive a subcutaneous ICD over a transvenous device.^16^ Furthermore, in a study in patients implanted with an atrial sensing dipole found that subclinical AF was diagnosed with an incidence of 13%, similar to dual chamber ICDs in the cohort.^17^ This is lower than our rate of 29%, which is likely driven by difference in comorbidities with the aforementioned study having a mean CHADS_2_‐VAS_C_ score of 2.1, while ours was 3.57.^17^

Dual chamber ICDs have traditionally been preferred over single chamber devices for better discrimination between ventricular and supraventricular arrhythmias, especially in patients not requiring pacing. The Detect Supraventricular Tachycardia Study showed that dual chamber detection was significantly better at detecting supraventricular arrhythmias compared to ventricular only detection.^18^ However, dual chamber ICDs increase risk of lead and generator‐related complications. Data from Matlock et al. and a large French ICD registry showed that dual chamber ICDs are significantly more likely to be associated with complications including perioperative lead dysfunction, pocket hematoma, and lead interference.^10^, ^19^ Adding an atrial lead also increases the rate of battery depletion and thus the need for more frequent generator replacement.

In this study, we demonstrated the real‐world use of the VBAF algorithm and subsequent management of patients with newly detected AF. What remains unknown and what requires further study is whether the physicians treating patients with anticoagulation improves outcomes and prevent stroke in these patients with newly detected AF on their VBAF enabled ICDs, and whether earlier intervention with antiarrhythmic drug therapy or cardiac ablation based on VBAF algorithm improves mortality or morbidity.

There are limitations associated with this study that should be acknowledged. First, this was a real‐world registry study and we are not able to discern why clinicians implanted the VBAF‐capable device over another other type of ICD. Second, the follow‐up duration of the study was only a mean of 22.5 months. Furthermore, for the blinded adjudication only a portion of each episode is stored in the device and therefore longer episodes not adjudicated as AF could have had AF later on during the episode and therefore missed as a true AF event. Also, patients with a high volume of episodes had older episodes overwritten and not available for review. Lastly, the device ability to detect an AF relies in the presence of an irregular ventricular response and may miss AF events with very rapid conduction to the ventricle where it regularizes and approaches the functional refractory period of the atrioventricular node and may miss regularized atrial flutter and tachycardia.

## 5 CONCLUSIONS

In a real‐world prospective registry with patients implanted with VBAF‐capable single chamber ICDs, the rates of previously undiagnosed AF episode are substantial and mirror those observed on previous dual chamber device studies. Continuous AF monitoring with the new VBAF algorithm permits early identification and actionable treatment for patients with undiagnosed AF. Future studies are needed to address the optimal management of patients with CIED‐detected AF.

## CONFLICTS OF INTEREST

A. R: Reports consulting fees and honoraria from Medtronic, Boston Scientific, and Philips, grants from Medtronic, and participation on an advisory board with Medtronic Bos. P. A. F: Reports licenses and patents with Anumana, Eko Health, Alive Cor, and Marani Health, advisory board participation at Anumana, a leadership position with xAI.health and MediCool, steering committee participation and advisory group participation at Boston Scientific and Medtronic, funds paid to their institution, and stock from MediCool, Marani Health, and xAI.health. J. A. M, M. L. B., and D. R. L: Employment Medtronic. B. L. W: Consultant for Medtronic, Abbott, and Philips. The remaining authors declare no conflict of interest.
